# (*S*_C_,*R*_S_)-Bromido­(*N*-{4-methyl-1-[(4-methyl­phenyl)sul­fan­yl]­pentan-2-yl}-*N′*-(pyridin-2-yl)imidazol-2-yl­idene)palladium(II) bromide

**DOI:** 10.1107/S2414314624003602

**Published:** 2024-04-26

**Authors:** Xianggui Zeng, Yanze Xu, Shuling Guo, Yongmei Xiao, Jinwei Yuan, Liangru Yang

**Affiliations:** a School of Chemistry & Chemical Engineering, Henan University of Technology, Zhengzhou 450001, People’s Republic of China; Purdue University, USA

**Keywords:** crystal structure, *N*-heterocyclic carbene, palladium(II), hydrogen bonds

## Abstract

The mol­ecule of the title NC_NHC_S pincer *N*-heterocyclic carbene palladium(II) complex, [PdBr(C_21_H_25_N_3_S)]Br, exhibits a slightly distorted square-planar coordination at the palladium(II) atom, with the five-membered chelate ring nearly planar. The six-membered chelate ring adopts an envelope conformation. Upon chelation, the sulfur atom becomes a stereogenic centre with an *R_S_* configuration induced by the chiral carbon of the precursor imidazolium salt. There are intra­molecular C—H⋯Br—Pd hydrogen bonds in the structure. The two inter­stitial Br atoms, as the counter-anion of the structure, are both located on crystallographic twofold axes and are connected to the complex cations *via* C—H⋯·Br hydrogen bonds.

## Abstract 2

The mol­ecule of the title NC_NHC_S pincer *N*-heterocyclic carbene palladium(II) complex, [PdBr(C_21_H_25_N_3_S)]Br, exhibits a slightly distorted square-planar coordination at the palladium(II) atom, with the five-membered chelate ring nearly planar. The six-membered chelate ring adopts an envelope conformation. Upon chelation, the sulfur atom becomes a stereogenic centre with an *R_S_* configuration induced by the chiral carbon of the precursor imidazolium salt.

## Graphical abstract

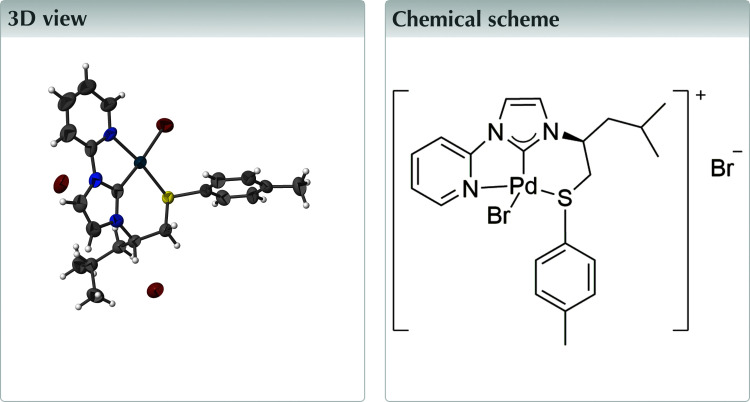

## Structure description

**Figure 1:**
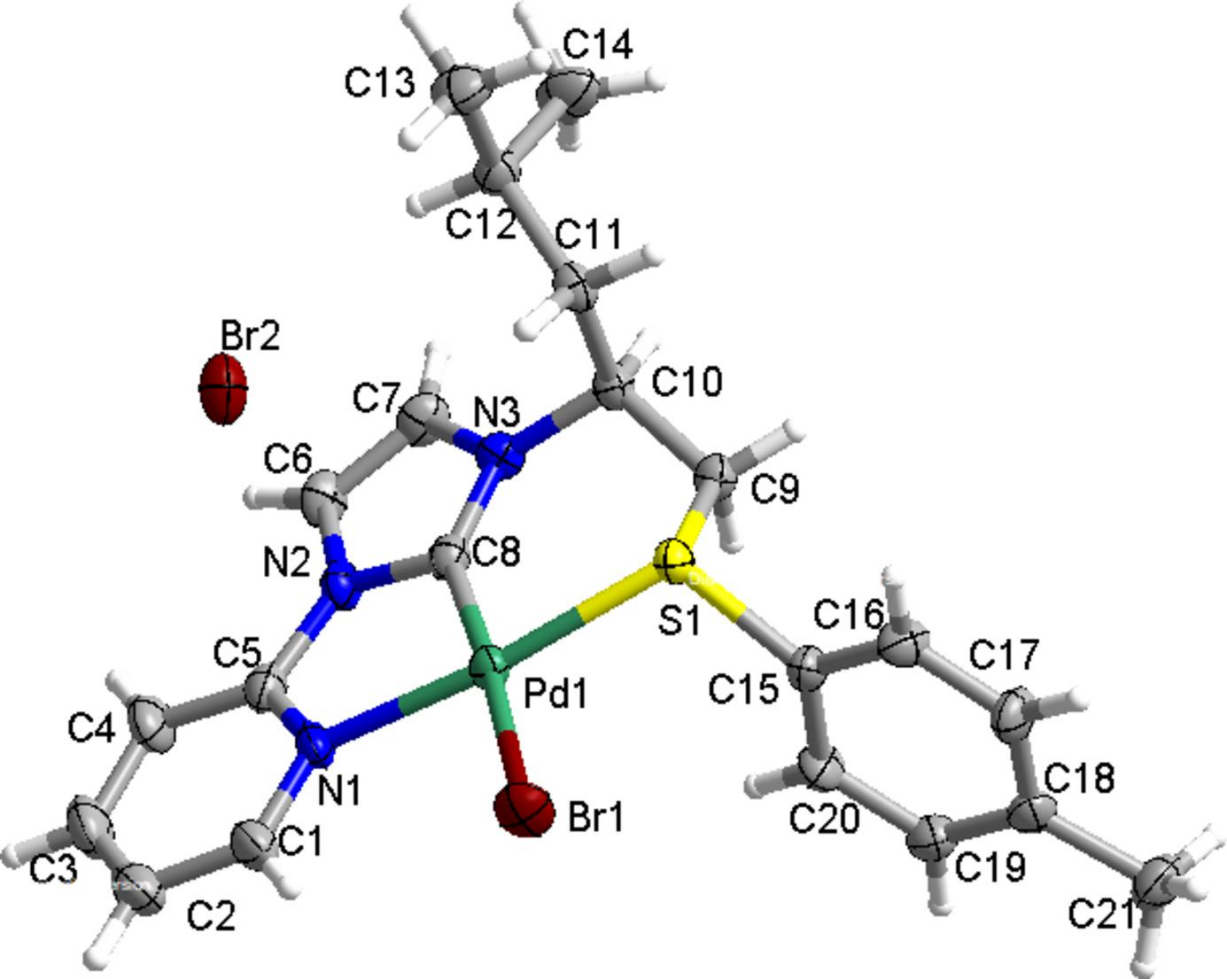
The mol­ecular structure of the title complex, shown with 50% probability displacement ellipsoids.

*N*-Heterocyclic carbenes (NHCs) have been widely used as ancillary ligands in coord­ination chemistry and organic catalysis due to their characteristic electronic properties and easy structural modification (Hopkinson *et al.*, 2014 ▸; Gardiner *et al.*, 2018 ▸). Introduction of a coordinating heteroatom functional group to the N-atom substituents of the NHCs leads to the formation of a potentially chelating ligand, and facilitates the formation of stable pincer NHC–metal complexes that can possess catalytic activities. Metal complexes containing heteroatom donors, such as P, N, O and S, have been synthesized, characterized and employed extensively as catalysts for a variety of organic transformations (Ahrens *et al.*, 2006 ▸; Bierenstiel & Cross, 2011 ▸; Meyer *et al.*, 2012 ▸; Peris & Crabtree, 2004 ▸). Our group has investigated the synthesis and catalytic performance of a series of chelating NHC–palladium complexes derived from natural amino alcohols (Yang *et al.*, 2015 ▸, 2023 ▸; Yang, Zhang, Xiao & Mao, 2016 ▸; Yang, Zhang, Yuan *et al.*, 2016 ▸; Meng *et al.*, 2022 ▸). As part of our work on the study of NHC–metal complexes containing heteroatom-functionalized N-atom substituents, we present here the crystal structure of the title NC_NHC_S pincer NHC palladium(II) complex (Fig. 1 ▸).

In the title complex, the palladium(II) atom is coordinated to C8, N1, Br1, and S1, resulting in a slightly distorted square-planar coordination. The Pd1—C8, Pd1—N1, Pd1—Br1 and Pd1—S1 bond lengths are 1.946 (8), 2.093 (6), 2.4663 (10), and 2.2603 (17) Å, respectively. The five-membered chelate ring (C8/Pd1/N1/C5/N2) is almost planar, with Pd1—N1—C5—N2 and C5—N2—C8—Pd1 torsion angles of −0.3 (8) and 2.0 (8)°, respectively. The six-membered chelate ring (C8/Pd1/S1/C9/C10/N3) adopts an envelope conformation with puckering parameters of θ = 51.6 (6)° and φ= 125.4 (8)°, which are close to the expected values for this conformation (Boeyens, 1978 ▸).

**Figure 2:**
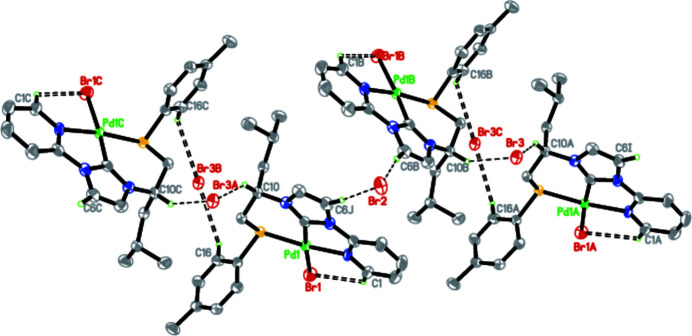
The C—H⋯Br inter­actions in the structure.

Upon chelation, the sulfur atom becomes a stereogenic centre, resulting in the formation of mol­ecules with an *R_S_* configuration. This can be attributed to the chiral induction of the chiral carbon C(5), which retains the same *S* configuration as in the precursor imidazolium salt. The environment of the sulfur atoms of the mol­ecule is approximately triangular pyramidal. This is indicated by the bond angles C9—S1—Pd1, C15—S1—Pd1 and C9—S1–15, which were found to be 106.2 (2), 111.0 (2), and 97.2 (3)°, respectively, with an average of 105.0°. In the crystal, intra- and inter­molecular C—H⋯Br hydrogen bonds occur (Table 1 ▸, Fig. 2 ▸).

## Synthesis and crystallization

A mixture of (*S*)-*N*-(4-methyl-1-(*p*-tolyl­thio)­pentan-2-yl)-*N′*-(pyridin-2-yl)-1*H*-imidazolium bromide (1.0 mmol, 0.43 g), PdCl_2_ (1.0 mmol, 0.18 g), NaOAc (1.0 mmol, 0.10 g), and NaBr (4 mmol, 0.41 g) in CH_3_CN (10 ml) was heated at 80°C for 24 h, and then the volatiles were evaporated. Purification of the residue by column chromatography (silica gel, CH_2_Cl_2_/MeOH 15/1 ∼1:1, *v*/*v*) produced the title complex as a yellow solid (0.32 g, 60%). Crystallization of the solid from CH_3_CN afforded the title complex as yellow crystals, m.p. 269–277°C. HR—MS (ESI) *m*/*z* calculated for C_21_H_25_BrN_3_PdS^+^ (*M* – Br)^+^ 535.9987, found 535.9998. F T–IR (ATR mode): ν = 3388, 3012, 2910, 1681, 1496, 1376, 1316, 1144, 1014, 914, 806, 780, 738, 666, 448 cm^−1.^ [α]^15^_589_: 8.3 (1.00, CH_2_Cl_2_).

## Refinement

Crystal data, data collection and structure refinement details are summarized in Table 2 ▸.

## Supplementary Material

Crystal structure: contains datablock(s) I. DOI: 10.1107/S2414314624003602/zl4070sup1.cif

Structure factors: contains datablock(s) I. DOI: 10.1107/S2414314624003602/zl4070Isup3.hkl

CCDC reference: 2312031

Additional supporting information:  crystallographic information; 3D view; checkCIF report

## Figures and Tables

**Table 1:** Hydrogen-bond geometry (Å, °)

*D*—H⋯*A*	*D*—H	H⋯*A*	*D*⋯*A*	*D*—H⋯*A*
C1—H1⋯Br1	0.93	2.90	3.510 (10)	124
C6—H6⋯Br2^i^	0.93	2.74	3.661 (9)	173
C10—H10⋯Br3	0.98	2.78	3.624 (7)	144
C16—H16⋯Br3^ii^	0.93	3.11	3.742 (7)	127

Symmetry codes: (i) 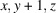 ; (ii) 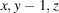 .

**Table 2:** Experimental details

Crystal data	
Chemical formula	[PdBr(C_21_H_25_N_3_S)]Br
*M*_r_	617.72
Crystal system, space group	Monoclinic, *C*2
Temperature (K)	293
*a*, *b*, *c* (Å)	25.8993 (7), 6.6206 (2), 13.4938 (3)
β (°)	96.425 (2)
*V* (Å^3^)	2299.23 (12)
*Z*	4
Radiation type	Cu *K*α
μ (mm^−1^)	11.52
Crystal size (mm)	0.14 × 0.1 × 0.03

Data collection	
Diffractometer	Xcalibur, Eos, Gemini
Absorption correction	Multi-scan (*CrysAlis PRO*; Rigaku OD, 2023 ▸)
*T*_min_, *T*_max_	0.419, 1.000
No. of measured, independent and observed [*I* > 2σ(*I*)] reflections	20069, 3950, 3693
*R*_int_	0.050
(sin θ/λ)_max_ (Å^−1^)	0.611

Refinement	
*R*[*F* ^2^ > 2σ(*F* ^2^)], *wR*(*F* ^2^), *S*	0.030, 0.072, 1.04
No. of reflections	3950
No. of parameters	258
No. of restraints	1
H-atom treatment	H-atom parameters constrained
Δρ_max_, Δρ_min_ (e Å^−3^)	0.36, −0.38
Absolute structure	Flack *x* determined using 1371 quotients [(*I* ^+^)−(*I* ^−^)]/[(*I* ^+^)+(*I* ^−^)] (Parsons *et al.*, 2013 ▸)
Absolute structure parameter	−0.030 (7)

Computer programs: *CrysAlis PRO* (Rigaku OD, 2023 ▸), *SHELXS* (Sheldrick, 2008 ▸), *SHELXL* (Sheldrick, 2015 ▸) and *OLEX2* (Dolomanov *et al.*, 2009 ▸).

## full crystallographic data

### Crystal data

**Table:**

[PdBr(C_21_H_25_N_3_S)]Br	*F*(000) = 1216
*M**_r_* = 617.72	*D*_x_ = 1.785 Mg m^−^^3^
Monoclinic, *C*2	Cu *K*α radiation, λ = 1.54184 Å
*a* = 25.8993 (7) Å	Cell parameters from 8595 reflections
*b* = 6.6206 (2) Å	θ = 4.5–70.1°
*c* = 13.4938 (3) Å	µ = 11.52 mm^−^^1^
β = 96.425 (2)°	*T* = 293 K
*V* = 2299.23 (12) Å^3^	Plate, colourless
*Z* = 4	0.14 × 0.1 × 0.03 mm

### Data collection

**Table:**

Xcalibur, Eos, Gemini diffractometer	3950 independent reflections
Radiation source: fine-focus sealed X-ray tube, Enhance (Cu) X-ray Source	3693 reflections with *I* > 2σ(*I*)
Graphite monochromator	*R*_int_ = 0.050
Detector resolution: 16.2312 pixels mm^-1^	θ_max_ = 70.5°, θ_min_ = 3.4°
ω scans	*h* = −31→31
Absorption correction: multi-scan (CrysAlisPro; Rigaku OD, 2023)	*k* = −8→6
*T*_min_ = 0.419, *T*_max_ = 1.000	*l* = −16→16
20069 measured reflections	

### Refinement

**Table:**

Refinement on *F*^2^	H-atom parameters constrained
Least-squares matrix: full	*w* = 1/[σ^2^(*F*_o_^2^) + (0.033*P*)^2^ + 2.7656*P*] where *P* = (*F*_o_^2^ + 2*F*_c_^2^)/3
*R*[*F*^2^ > 2σ(*F*^2^)] = 0.030	(Δ/σ)_max_ < 0.001
*wR*(*F*^2^) = 0.072	Δρ_max_ = 0.36 e Å^−^^3^
*S* = 1.04	Δρ_min_ = −0.38 e Å^−^^3^
3950 reflections	Extinction correction: SHELXL-2014/7 (Sheldrick, 2015), Fc^*^=kFc[1+0.001xFc^2^λ^3^/sin(2θ)]^-1/4^
258 parameters	Extinction coefficient: 0.00034 (4)
1 restraint	Absolute structure: Flack *x* determined using 1371 quotients [(*I*^+^)-(*I*^-^)]/[(*I*^+^)+(*I*^-^)] (Parsons *et al.*, 2013)
Primary atom site location: structure-invariant direct methods	Absolute structure parameter: −0.030 (7)
Hydrogen site location: inferred from neighbouring sites	

### Special details

**Table:**

Geometry. All esds (except the esd in the dihedral angle between two l.s. planes) are estimated using the full covariance matrix. The cell esds are taken into account individually in the estimation of esds in distances, angles and torsion angles; correlations between esds in cell parameters are only used when they are defined by crystal symmetry. An approximate (isotropic) treatment of cell esds is used for estimating esds involving l.s. planes.
Refinement. The H atoms on the carbons were positioned geometrically and constrained to ride on their parent atoms. C—H bond distances were constrained to 0.93 Å for aromatic and alkene C—H moieties, and to 0.98, 0.91 and 0.96 Å for aliphatic C—H, CH_2_ and CH_3_ moieties, respectively. Methyl CH_3_ H atoms were allowed to rotate but not to tip to best fit the experimental electron density. *U*_iso_(H) values were set to a multiple of *U*_eq_(C) with 1.5 for CH_3_, and 1.2 for C—H and CH_2_ units, respectively.

### Fractional atomic coordinates and isotropic or equivalent isotropic displacement parameters (Å^2^)

**Table:**

	*x*	*y*	*z*	*U*_iso_*/*U*_eq_	
Pd1	0.37598 (2)	0.06095 (8)	0.28758 (3)	0.03701 (15)	
S1	0.40637 (6)	−0.0902 (3)	0.15555 (12)	0.0381 (4)	
N1	0.3574 (2)	0.2356 (11)	0.4079 (4)	0.0443 (16)	
N2	0.4255 (2)	0.4165 (10)	0.3607 (4)	0.0413 (14)	
N3	0.4680 (2)	0.3203 (10)	0.2419 (4)	0.0381 (13)	
C1	0.3187 (3)	0.2026 (16)	0.4635 (6)	0.057 (2)	
H1	0.2972	0.0912	0.4501	0.069*	
C2	0.3100 (4)	0.3329 (18)	0.5414 (7)	0.069 (3)	
H2	0.2832	0.3085	0.5804	0.083*	
C3	0.3414 (4)	0.4954 (18)	0.5587 (7)	0.074 (3)	
H3	0.3359	0.5840	0.6099	0.089*	
C4	0.3813 (3)	0.5318 (18)	0.5021 (6)	0.061 (2)	
H4	0.4033	0.6422	0.5144	0.073*	
C5	0.3873 (3)	0.3975 (13)	0.4263 (5)	0.0450 (18)	
C6	0.4650 (3)	0.5562 (15)	0.3556 (5)	0.0478 (16)	
H6	0.4719	0.6686	0.3963	0.057*	
C7	0.4911 (3)	0.4978 (13)	0.2812 (6)	0.049 (2)	
H7	0.5195	0.5632	0.2593	0.059*	
C8	0.4280 (2)	0.2737 (13)	0.2911 (5)	0.0393 (16)	
C9	0.4434 (2)	0.1033 (12)	0.0964 (5)	0.0415 (18)	
H9A	0.4574	0.0439	0.0394	0.050*	
H9B	0.4199	0.2108	0.0720	0.050*	
C10	0.4877 (3)	0.1938 (11)	0.1649 (5)	0.0366 (15)	
H10	0.5072	0.2821	0.1242	0.044*	
C11	0.5255 (2)	0.0360 (13)	0.2122 (5)	0.0397 (16)	
H11A	0.5325	−0.0599	0.1610	0.048*	
H11B	0.5085	−0.0373	0.2617	0.048*	
C12	0.5777 (3)	0.1161 (12)	0.2623 (5)	0.0441 (19)	
H12	0.5707	0.2109	0.3150	0.053*	
C13	0.6081 (3)	−0.0612 (16)	0.3099 (7)	0.065 (3)	
H13A	0.5892	−0.1208	0.3598	0.097*	
H13B	0.6413	−0.0150	0.3404	0.097*	
H13C	0.6132	−0.1599	0.2598	0.097*	
C14	0.6090 (3)	0.2258 (18)	0.1894 (7)	0.072 (3)	
H14A	0.6092	0.1467	0.1298	0.108*	
H14B	0.6440	0.2448	0.2196	0.108*	
H14C	0.5935	0.3549	0.1729	0.108*	
C15	0.3558 (3)	−0.1214 (13)	0.0549 (5)	0.0428 (18)	
C16	0.3572 (3)	−0.2944 (13)	−0.0012 (6)	0.0494 (19)	
H16	0.3814	−0.3949	0.0174	0.059*	
C17	0.3221 (3)	−0.3171 (14)	−0.0853 (6)	0.054 (2)	
H17	0.3225	−0.4353	−0.1224	0.064*	
C18	0.2862 (3)	−0.1665 (14)	−0.1157 (5)	0.048 (2)	
C19	0.2848 (3)	0.0021 (14)	−0.0560 (5)	0.051 (2)	
H19	0.2601	0.1015	−0.0734	0.061*	
C20	0.3190 (3)	0.0263 (14)	0.0282 (5)	0.049 (2)	
H20	0.3176	0.1415	0.0672	0.059*	
C21	0.2512 (3)	−0.1845 (17)	−0.2126 (6)	0.067 (3)	
H21A	0.2684	−0.1290	−0.2659	0.101*	
H21B	0.2195	−0.1116	−0.2075	0.101*	
H21C	0.2433	−0.3242	−0.2261	0.101*	
Br1	0.30979 (4)	−0.20451 (15)	0.29589 (7)	0.0603 (3)	
Br2	0.5000	0.0140 (2)	0.5000	0.0609 (4)	
Br3	0.5000	0.61892 (17)	0.0000	0.0480 (3)	

### Atomic displacement parameters (Å^2^)

**Table:**

	*U*^11^	*U*^22^	*U*^33^	*U*^12^	*U*^13^	*U*^23^
Pd1	0.0397 (2)	0.0390 (3)	0.0327 (2)	−0.0004 (2)	0.00594 (15)	−0.0005 (2)
S1	0.0391 (8)	0.0388 (11)	0.0362 (8)	0.0001 (7)	0.0032 (6)	−0.0042 (8)
N1	0.050 (3)	0.054 (5)	0.030 (3)	0.012 (3)	0.009 (2)	−0.007 (3)
N2	0.050 (3)	0.036 (4)	0.038 (3)	0.007 (3)	0.003 (2)	−0.007 (3)
N3	0.042 (3)	0.031 (4)	0.042 (3)	−0.003 (3)	0.006 (2)	0.002 (3)
C1	0.054 (4)	0.073 (7)	0.047 (4)	0.009 (4)	0.015 (3)	−0.002 (4)
C2	0.063 (5)	0.091 (9)	0.055 (5)	0.018 (5)	0.019 (4)	0.003 (5)
C3	0.089 (7)	0.087 (9)	0.050 (4)	0.020 (6)	0.019 (4)	−0.017 (5)
C4	0.071 (5)	0.058 (7)	0.054 (4)	0.012 (5)	0.010 (3)	−0.019 (5)
C5	0.051 (4)	0.043 (5)	0.038 (4)	0.010 (4)	−0.006 (3)	−0.007 (4)
C6	0.061 (4)	0.030 (4)	0.050 (4)	−0.001 (4)	−0.001 (3)	−0.008 (4)
C7	0.052 (4)	0.038 (5)	0.055 (4)	−0.011 (3)	0.003 (3)	0.000 (4)
C8	0.042 (3)	0.037 (5)	0.036 (3)	0.005 (3)	−0.003 (3)	−0.002 (3)
C9	0.042 (3)	0.047 (6)	0.035 (3)	0.001 (3)	0.003 (2)	−0.001 (3)
C10	0.045 (3)	0.031 (4)	0.034 (3)	−0.002 (3)	0.008 (3)	−0.001 (3)
C11	0.049 (3)	0.032 (4)	0.039 (3)	0.002 (3)	0.009 (2)	0.000 (4)
C12	0.043 (3)	0.048 (6)	0.041 (3)	−0.005 (3)	0.004 (3)	−0.001 (3)
C13	0.051 (4)	0.073 (7)	0.068 (5)	0.007 (4)	−0.005 (4)	0.010 (5)
C14	0.051 (5)	0.096 (9)	0.069 (6)	−0.019 (5)	0.006 (4)	0.022 (6)
C15	0.038 (3)	0.051 (5)	0.039 (3)	−0.001 (3)	0.003 (3)	−0.009 (4)
C16	0.047 (4)	0.038 (5)	0.062 (5)	0.003 (3)	0.001 (3)	−0.009 (4)
C17	0.056 (4)	0.051 (6)	0.051 (4)	−0.001 (4)	−0.006 (3)	−0.020 (4)
C18	0.036 (3)	0.058 (6)	0.048 (4)	−0.011 (3)	0.004 (3)	−0.006 (4)
C19	0.042 (4)	0.060 (7)	0.050 (4)	0.007 (3)	0.002 (3)	0.000 (4)
C20	0.051 (4)	0.046 (6)	0.051 (4)	0.014 (4)	0.004 (3)	−0.011 (4)
C21	0.058 (5)	0.079 (8)	0.061 (5)	−0.003 (5)	−0.014 (4)	−0.006 (5)
Br1	0.0612 (5)	0.0547 (7)	0.0674 (5)	−0.0142 (4)	0.0173 (4)	0.0042 (5)
Br2	0.0953 (9)	0.0421 (9)	0.0444 (5)	0.000	0.0037 (5)	0.000
Br3	0.0607 (6)	0.0392 (8)	0.0460 (5)	0.000	0.0141 (4)	0.000

### Geometric parameters (Å, º)

**Table:**

Pd1—S1	2.2603 (17)	C10—H10	0.9800
Pd1—N1	2.093 (6)	C10—C11	1.522 (10)
Pd1—C8	1.946 (8)	C11—H11A	0.9700
Pd1—Br1	2.4663 (10)	C11—H11B	0.9700
S1—C9	1.836 (7)	C11—C12	1.537 (9)
S1—C15	1.789 (7)	C12—H12	0.9800
N1—C1	1.335 (9)	C12—C13	1.517 (11)
N1—C5	1.330 (10)	C12—C14	1.527 (10)
N2—C5	1.405 (9)	C13—H13A	0.9600
N2—C6	1.387 (10)	C13—H13B	0.9600
N2—C8	1.340 (9)	C13—H13C	0.9600
N3—C7	1.396 (10)	C14—H14A	0.9600
N3—C8	1.328 (9)	C14—H14B	0.9600
N3—C10	1.470 (9)	C14—H14C	0.9600
C1—H1	0.9300	C15—C16	1.376 (11)
C1—C2	1.397 (13)	C15—C20	1.384 (11)
C2—H2	0.9300	C16—H16	0.9300
C2—C3	1.354 (15)	C16—C17	1.380 (11)
C3—H3	0.9300	C17—H17	0.9300
C3—C4	1.373 (12)	C17—C18	1.393 (12)
C4—H4	0.9300	C18—C19	1.379 (11)
C4—C5	1.376 (11)	C18—C21	1.511 (10)
C6—H6	0.9300	C19—H19	0.9300
C6—C7	1.329 (10)	C19—C20	1.370 (10)
C7—H7	0.9300	C20—H20	0.9300
C9—H9A	0.9700	C21—H21A	0.9600
C9—H9B	0.9700	C21—H21B	0.9600
C9—C10	1.513 (9)	C21—H21C	0.9600
			
S1—Pd1—Br1	91.46 (5)	C9—C10—H10	107.3
N1—Pd1—S1	170.64 (19)	C9—C10—C11	113.1 (6)
N1—Pd1—Br1	97.90 (19)	C11—C10—H10	107.3
C8—Pd1—S1	92.2 (2)	C10—C11—H11A	108.3
C8—Pd1—N1	78.4 (3)	C10—C11—H11B	108.3
C8—Pd1—Br1	175.9 (2)	C10—C11—C12	116.1 (7)
C9—S1—Pd1	106.2 (2)	H11A—C11—H11B	107.4
C15—S1—Pd1	111.0 (2)	C12—C11—H11A	108.3
C15—S1—C9	97.2 (3)	C12—C11—H11B	108.3
C1—N1—Pd1	126.7 (6)	C11—C12—H12	108.5
C5—N1—Pd1	114.3 (5)	C13—C12—C11	108.0 (7)
C5—N1—C1	119.0 (7)	C13—C12—H12	108.5
C6—N2—C5	131.8 (6)	C13—C12—C14	110.6 (7)
C8—N2—C5	118.1 (7)	C14—C12—C11	112.7 (6)
C8—N2—C6	110.0 (6)	C14—C12—H12	108.5
C7—N3—C10	125.5 (6)	C12—C13—H13A	109.5
C8—N3—C7	109.3 (6)	C12—C13—H13B	109.5
C8—N3—C10	124.9 (6)	C12—C13—H13C	109.5
N1—C1—H1	119.5	H13A—C13—H13B	109.5
N1—C1—C2	121.1 (9)	H13A—C13—H13C	109.5
C2—C1—H1	119.5	H13B—C13—H13C	109.5
C1—C2—H2	120.8	C12—C14—H14A	109.5
C3—C2—C1	118.4 (8)	C12—C14—H14B	109.5
C3—C2—H2	120.8	C12—C14—H14C	109.5
C2—C3—H3	119.4	H14A—C14—H14B	109.5
C2—C3—C4	121.3 (9)	H14A—C14—H14C	109.5
C4—C3—H3	119.4	H14B—C14—H14C	109.5
C3—C4—H4	121.5	C16—C15—S1	116.9 (6)
C3—C4—C5	117.0 (10)	C16—C15—C20	120.2 (7)
C5—C4—H4	121.5	C20—C15—S1	122.7 (6)
N1—C5—N2	113.0 (6)	C15—C16—H16	120.4
N1—C5—C4	123.2 (8)	C15—C16—C17	119.2 (8)
C4—C5—N2	123.8 (8)	C17—C16—H16	120.4
N2—C6—H6	126.8	C16—C17—H17	119.3
C7—C6—N2	106.5 (7)	C16—C17—C18	121.4 (8)
C7—C6—H6	126.8	C18—C17—H17	119.3
N3—C7—H7	126.3	C17—C18—C21	121.0 (8)
C6—C7—N3	107.4 (7)	C19—C18—C17	117.9 (7)
C6—C7—H7	126.3	C19—C18—C21	121.1 (8)
N2—C8—Pd1	116.1 (5)	C18—C19—H19	119.3
N3—C8—Pd1	137.2 (6)	C20—C19—C18	121.5 (8)
N3—C8—N2	106.7 (7)	C20—C19—H19	119.3
S1—C9—H9A	108.7	C15—C20—H20	120.1
S1—C9—H9B	108.7	C19—C20—C15	119.7 (8)
H9A—C9—H9B	107.6	C19—C20—H20	120.1
C10—C9—S1	114.1 (5)	C18—C21—H21A	109.5
C10—C9—H9A	108.7	C18—C21—H21B	109.5
C10—C9—H9B	108.7	C18—C21—H21C	109.5
N3—C10—C9	111.0 (5)	H21A—C21—H21B	109.5
N3—C10—H10	107.3	H21A—C21—H21C	109.5
N3—C10—C11	110.6 (5)	H21B—C21—H21C	109.5
			
Pd1—S1—C9—C10	58.1 (5)	C7—N3—C8—Pd1	−179.7 (6)
Pd1—S1—C15—C16	−143.9 (5)	C7—N3—C8—N2	−0.4 (8)
Pd1—S1—C15—C20	41.2 (7)	C7—N3—C10—C9	−144.6 (7)
Pd1—N1—C1—C2	−179.1 (6)	C7—N3—C10—C11	89.0 (8)
Pd1—N1—C5—N2	−0.3 (8)	C8—N2—C5—N1	−1.1 (9)
Pd1—N1—C5—C4	179.8 (6)	C8—N2—C5—C4	178.8 (8)
S1—C9—C10—N3	−69.7 (7)	C8—N2—C6—C7	0.8 (9)
S1—C9—C10—C11	55.2 (7)	C8—N3—C7—C6	0.9 (9)
S1—C15—C16—C17	−173.9 (6)	C8—N3—C10—C9	41.5 (9)
S1—C15—C20—C19	173.0 (6)	C8—N3—C10—C11	−84.8 (8)
N1—C1—C2—C3	0.7 (14)	C9—S1—C15—C16	105.7 (6)
N2—C6—C7—N3	−1.0 (9)	C9—S1—C15—C20	−69.3 (7)
N3—C10—C11—C12	−68.7 (7)	C9—C10—C11—C12	166.1 (5)
C1—N1—C5—N2	−178.3 (7)	C10—N3—C7—C6	−173.7 (7)
C1—N1—C5—C4	1.8 (12)	C10—N3—C8—Pd1	−5.0 (11)
C1—C2—C3—C4	−0.5 (15)	C10—N3—C8—N2	174.3 (6)
C2—C3—C4—C5	0.9 (15)	C10—C11—C12—C13	176.6 (6)
C3—C4—C5—N1	−1.6 (13)	C10—C11—C12—C14	−60.9 (9)
C3—C4—C5—N2	178.6 (8)	C15—S1—C9—C10	172.5 (5)
C5—N1—C1—C2	−1.3 (12)	C15—C16—C17—C18	1.5 (13)
C5—N2—C6—C7	177.4 (7)	C16—C15—C20—C19	−1.8 (12)
C5—N2—C8—Pd1	2.0 (8)	C16—C17—C18—C19	−3.5 (12)
C5—N2—C8—N3	−177.4 (6)	C16—C17—C18—C21	174.6 (8)
C6—N2—C5—N1	−177.5 (7)	C17—C18—C19—C20	2.9 (12)
C6—N2—C5—C4	2.4 (13)	C18—C19—C20—C15	−0.3 (12)
C6—N2—C8—Pd1	179.2 (5)	C20—C15—C16—C17	1.2 (12)
C6—N2—C8—N3	−0.2 (8)	C21—C18—C19—C20	−175.2 (8)

### Hydrogen-bond geometry (Å, º)

**Table:**

*D*—H···*A*	*D*—H	H···*A*	*D*···*A*	*D*—H···*A*
C1—H1···Br1	0.93	2.90	3.510 (10)	124
C6—H6···Br2^i^	0.93	2.74	3.661 (9)	173
C10—H10···Br3	0.98	2.78	3.624 (7)	144
C16—H16···Br3^ii^	0.93	3.11	3.742 (7)	127

Symmetry codes: (i) *x*, *y*+1, *z*; (ii) *x*, *y*−1, *z*.
